# Stimulus reconstruction based on postsynaptic potentials of leech interneurons

**DOI:** 10.1186/1471-2202-12-S1-P179

**Published:** 2011-07-18

**Authors:** Markus Kappel, Friederice Pirschel, Jutta Kretzberg

**Affiliations:** 1 Computational Neuroscience, Institute of Biology and Environmental Sciences, University of Oldenburg, D-26111 Oldenburg, Germany

## 

Most studies on the neuronal encoding of sensory stimuli have been performed based on spike responses. The classically considered neuronal response features like spike count, spike timing or ISIs rely on the all-or-nothing characteristic of spike responses. However, little is known about stimulus encoding on the level of integrated postsynaptic responses. In particular, no standard methods exist to analyze stimulus encoding with graded potentials. Moreover, the huge amount of stimulus-independent fluctuations present in intracellularly recorded membrane potentials complicates the analysis of graded responses.

In this study, we use interneurons of the medical leech to analyze stimulus encoding based on graded membrane potential fluctuations. The local bend network controls the reflex behavior of the leech bending away when the skin is touched lightly [1]. It consists of four sensory P (‘pressure’) cells, approximately 20 interneurons and four groups of motorneurons, all of which can be identified individually. EPSPs induced by P cell spikes can be recorded intracellularly from the soma of interneurons, where spike amplitudes are small (<7mV) [2]. In a related study (see Pirschel & Kretzberg, this issue) we investigated the encoding of tactile stimulus parameters by P cell spike responses. We found that the intensity of pressure applied to the skin influences the spike count as well as the length of the 1^st^ interspike interval (ISI) and both response parameters yielded similar stimulus reconstruction performances. In this sense, we have analyzed the influence of presynaptic P cell spike count and 1^st^ ISI on postsynaptic interneuron responses. Precisely timed P cell spikes were elicited in intracellular double recordings in five cell pairs with short pulses of current injection. We simulated tactile skin stimulation with three pressure intensities (≥6 presentations each) by inducing previously determined typical P cell spike responses, differing in spike count and ISI lengths. For comparison, we used protocols in which only the spike count or only the 1^st^ ISI of P cell spikes were varied. Stimulus reconstruction based on interneuron recordings was performed with a maximum likelihood approach, using the area under the EPSP, the EPSP slope, or the EPSP maximum amplitude.

When using the EPSP area and an optimal time window of ~100-300ms for stimulus reconstruction, the three tactile stimuli simulated by realistic P cell spike patterns were reconstructed correctly in ~70% of the trials (chance level 33.3%). Interestingly, P cell responses differing only in their 1^st^ ISI could be discriminated faster (optimal time window 90-155ms) and more reliably (up to 90% correct). However, P cell responses differing only in spike count required long time windows (>350ms) and reached lower reconstruction performance (~50% correct). Using the EPSP slope yielded similar reconstruction performances and optimal time scales, but turned out to be more vulnerable to noisy data. Estimation based on maximum EPSP amplitude led to considerably lower performances.

## Conclusions

1. The area under the interneuron EPSP is a suitable responses feature for stimulus reconstruction.

2. Interneuron EPSPs in the leech local bend network depend more strongly on the 1^st^ ISI than on the spike count of presynaptic P cell responses.
